# Memory Modification and Authenticity: A Narrative Approach

**DOI:** 10.1007/s12152-022-09489-9

**Published:** 2022-02-16

**Authors:** Muriel Leuenberger

**Affiliations:** Oxford Uehiro Centre for Practical Ethics, Faculty of Philosophy, University of Oxford, Suite 8 Littlegate House, 16/17 St Ebbe’s Street, Oxford, OX1 1PT UK

**Keywords:** Memory modification, Narrative self, Identity authenticity

## Abstract

The potential of memory modification techniques (MMTs) has raised concerns and sparked a debate in neuroethics, particularly in the context of identity and authenticity. This paper addresses the question whether and how MMTs influence authenticity. I proceed by drawing two distinctions within the received views on authenticity. From this, I conclude that an analysis of MMTs based on a dual-basis, process view of authenticity is warranted, which implies that the influence of MMTs on authenticity crucially depends on the specifics of how memory modification would eventually work. Therefore, I continue with a systematic analysis of possible properties of MMTs in which I distinguish between the dimensions of memories and the kinds of experiences that can be modified as well as the properties of the process of memory modification. The impact of MMTs on authenticity is analyzed regarding the possible properties of MMTs and based on a narrative approach to authenticity which fulfills the requirements of a dual-basis, process view of authenticity. Lastly, I explore the potential of MMTs to shift the balance between self-discovery and self-creation within authenticity and thereby alter the concept itself as well as the value of authenticity.

## Introduction

The potential of memory modification techniques (MMTs) has raised concerns and sparked a heated debate in neuroethics. A commonly raised issue is that memory modification may impact identity and authenticity [1–8]. Memories are at the heart of our identity. They connect us to our past and inform us about who we are and how we ended up being who we are. According to the narrative identity view, your memories are the basis of your self-narrative, an internalized, evolving story of your life, which constitutes your personal identity.^1^ By deliberately manipulating our memories, we are manipulating ourselves. This prospect raises questions about authenticity. Can I be authentic if I erase uncomfortable memories of my past? What if I only lower their emotional impact? In this paper, I address these questions based on a narrative account of authenticity and through a systematic analysis of how MMTs could look like. Moreover, I explore how techniques like memory modification may alter the concept of authenticity itself as well as some possible consequences this may bring about.

The potential benefits of modifying memories range from memory enhancement to treatments of memory disorders, addiction, anxiety, and Post-Traumatic Stress Disorder (PTSD) [9, 8, 10]. Memory modification could be used to enhance, erase, dampen (i.e., lowering the emotional impact), or edit memories (i.e., replace with or add new elements). There are two possible time windows for memory modification. Either storage of the memory is modified (consolidation) by intervening before or shortly after an event, or the modification occurs in the liable state after retrieval (reconsolidation) [10]. So far, no method allows for a robust and easily implemented editing of unique human memories [10]. But the rapid progress in the science of memory suggests that the future development of successful MMTs is probable [10]. Therefore, potential ethical dangers and opportunities of MMTs should be addressed in a timely manner.

Various possible techniques for memory modification are being developed or are already in use. Pharmaceutics – notably the beta-blocker Propranolol, psychological methods, and optogenetics have shown particularly promising results so far. Propranolol blocks the effect of stress hormones that usually enhance memory consolidation of emotional events [9]. If taken shortly after or before the event, it has been able to reduce the stress response of memories without interfering with explicit, declarative, or autobiographical memory [9, 11–14]. It was however not possible to replicate the effect when Propranolol was administered later, during memory reconsolidation [15, 16]. Psychological methods can target reconsolidation but they have the disadvantage that they are often unreliable and prone to relapse [10]. They rely on, for example, overwriting a fear memory with a new safety memory (extinction training) [17] or updating the memory with new information (imagery rescripting) [18]. Optogenetics uses light to control the activation and deactivation of individual, genetically modified neurons [8]. Optogenetics for memory modification has so far only been conducted in animals. In animal models, it is possible to switch selected memories on and off, modify details of memories, manipulate the memory valence, or regain access to forgotten memories [8, 19–22]. It is a matter of controversy whether it will ever be safe to use in humans and whether it will be possible to target rich, autobiographic memories [23–26].

The contributions in the neuroethical literature on MMTs tend to discuss memory modification predominantly in terms of thought experiments, specific methods for memory modification, or a combination of the two. Commonly used thought experiments are Liz, as introduced by Alexandre Erler [1], who uses MMTs to cope with her memories of how she was bullied at school [4, 8, 27–29] or Lady Macbeth who modifies her memories of how she contributed to the murder of the king [1, 3, 7, 30–32]. Regarding specific methods for memory modification, there are lively discussions on the ethical implications of memory modification through Propranolol [2, 4, 7, 31] or optogenetics [8, 24, 26, 25]. Both routes, thought experiments as well as existing methods for memory modification, can provide valuable insights, but they do have limitations. Thought experiments focus on specific scenarios which are not feasible now and maybe never will be. The situation will likely be different or more complicated than the thought experiment suggests and relevant factors may be overlooked. The analysis of an existing method for memory modification may be closer to reality, but a lot of relevant information to make an ethical assessment (e.g., regarding their impact on authenticity), is not available yet. Crucially, information on how patients experience the modified memories is still either scarce or non-existent. As mentioned above, memory modification via optogenetics has so far only been tested on animals. In the case of Propranolol, human trials have been conducted and more information is available but many aspects regarding the phenomenology of modified memories or what kind of memories will likely be modified in the future are still unclear.

In this paper, I propose a third option to analyze the ethical implications of MMTs: by starting with a systematic analysis of possible properties of memory modification to then evaluate their impact on authenticity.^2^ By laying out the different ways how patients may experience modified memories, the kind of experiences that could be modified, and what the process of memory modification may look like, the set of considered parameters is broadened in comparison to thought experiments and specific methods. Thereby, the impact of MMTs on authenticity can be understood regarding both, MMTs which are currently available and methods which may be available in the future. Moreover, this analysis can suggest which kind of empirical studies would be helpful to assess the influence of MMTs on authenticity and how MMTs may be modified to reduce a negative impact on authenticity or promote positive effects.

A further novel contribution of this paper to the debate is that I use a dual-basis process view of authenticity based on a narrative concept of the self to analyze the influence of MMTs on authenticity. As I will argue, a dual-basis process account is less prone to making controversial metaphysical and empirical claims compared to conservation views or accounts centered around either self-discovery or self-creation while being able to capture some paradigmatic cases of authenticity. An ethical assessment of MMTs in terms of a dual-basis process view of authenticity can provide novel insights into potential threats and chances MMTs offer for authenticity which complement the existing literature. Moreover, the account is based on a narrative concept of the self, which is theoretically and empirically well established [33–35] while avoiding blurring the differences between narrative identity and narrative authenticity.

To address the question of how MMTs may impact authenticity, I start by drawing two distinctions within the variety of possible approaches to authenticity and argue for a dual-basis process view (part 2). From this, I conclude that the influence of MMTs on authenticity depends on the specifics of how MMTs may eventually work. Therefore, I continue with a systematic analysis of the possible properties of MMTs (part 3). Some of the properties I point out in this section have been discussed in the neuroethical literature before, for example, that MMTs may be used to modify formative memories [8]. The discussion on the possible dimensions of memory which may be modified (the factual content, the sensory-like experience, and the autonoetic consciousness) and the distinction in dimensions of memories, kinds of experiences, and properties of the process of memory modification are novel contributions to the debate. In the next part (4), I briefly introduce a narrative account of authenticity which is a dual-basis, process view of authenticity. Next, the impact of MMTs on narrative authenticity is analyzed regarding the previously discussed possible properties of MMTs (part 5). Before the conclusion, I elaborate on the potential of MMTs to alter the ideal of authenticity itself by expanding what we can change about ourselves (part 6).

## Authenticity: Two Distinctions

In the neuroethical literature, authenticity has recently been the subject of much debate. In particular, the potential of Deep Brain Stimulation to alter the personality of patients raised concerns about authenticity [36–40]. Authors have usually distinguished between authenticity as self-discovery and authenticity as self-creation (also referred to as essentialist and existentialist accounts) [41–43].^3^ According to a self-discovery view, being authentic entails finding and realizing the “true self”, a fixed, individual essence [44, 45]. In contrast, a self-creation-based approach understands authenticity as a matter of facing up to one’s possibilities through free choice and self-creation [46]. Individuals should free themselves from the demands of alleged essences or social norms to create themselves authentically. Both views are rarely anymore fully adopted in this stark form. Attenuated versions are still in use, such as Erler’s true self view [1] or Harry Frankfurt’s wholeheartedness approach [47] which can be understood as a version of a self-creation view and which is also prevalent in the neuroethical debate [1, 4, 5, 8, 37]. An essentialist view on authenticity is a common concept of folk understanding [48] and it seems to have functional merits [49]. However, it makes empirically and metaphysically problematic assumptions, notably that we have an individual essence “deep down” that we can discover and that remains untouched by external influences [50, 51]. Self-creation-centered views face similar problems (the notion of a radically free choice seems particularly problematic) [52] and they have been criticized for lacking practical utility [53]. In their place, dual-basis views have been introduced, combining elements of both [37, 42, 54–56]. A dual-basis account, balancing the possibilities and constraints of self-discovery and self-creation, seems most plausible and suited to reflect not only the complexity of human psychology but also the intricacy and ambivalence of the concept of authenticity [54, 57].

Elsewhere, I argue that another distinction can further map the field of received views on authenticity: a distinction between a *process view* of authenticity and a *conservation view* [58], see also Jonathan Pugh [59] for a similar distinction. A conservation view judges the authenticity of a person based on whether a certain set of characteristics is preserved. A person is authentic after memory modification as long as, for example, their core values or the characteristics they identify with remain unchanged. Authenticity hinges on the *content* of the change. On the other hand, according to a process view, we should judge authenticity based on the properties of the *process* of change. Memory modification is authentic if, for instance, it results from a free choice or if it promotes self-knowledge. It does not matter which aspects of the self change over time or to what degree, as long as the way in which they change is authentic.^4^ Authenticity as self-discovery is a conservation view whereas the self-creation account is a process view.^5^

Conservation views are common in folk psychology [48, 60]. However, they face the problem that they would encourage some people to preserve morally questionable characteristics. According to a conservationist ideal of authenticity, a thoroughly bad person should not change him- or herself. In this case, authenticity no longer seems like a particularly desirable ideal. Studies in experimental philosophy point to a way how in folk reasoning people try to avoid this consequence. The true self is perceived as positive and morally good whereas negative and undesirable traits are seen as less defining and part of the peripheral self [48, 60]. Positive personal changes are seen as discoveries of the true self and not as deviations from a morally questionable true self. However, as Nina Strohminger et al. have pointed out [48], this view is radically subjective since the true self depends on the values of the observer and it is unverifiable since even those who are consistently deplorable are deemed good “deep down”. Process views, on the other hand, avoid the dilemma of either supporting the preservation of deplorable characteristics or holding the radically subjective and unverifiable claim that we are all essentially good.^6^

Moreover, the process view seems to be able to capture paradigmatic cases which are fundamental to accounts of authenticity throughout history (e.g., by Rousseau or Sartre) and which still shape our intuitions: that externally imposed aspects of the self are a paradigmatic threat to authenticity. Beliefs, preferences, values, characteristics, and actions which have been acquired, for example, through manipulation, hypnotization, or brainwashing, are deemed inauthentic—not because they are not in line with the true self but because of how they were brought about, because of the *process* that caused the change. The question which aspects of the self are being changed through such strong external influences seems irrelevant to judge whether the change is authentic. If you have been hypnotized to dislike apples, this part of your personality is inauthentic, even if it is far from being a central element of who you are. This example implies that for authenticity, *how* we change matters over *what* changes.

Because the concept of authenticity has been adapted and shaped by various traditions, many and sometimes contradictory intuitions on what is and is not authentic are in circulation. It seems hardly possible to capture them all in a single account. With the account I propose, I aim at a conceptually and empirically plausible and coherent account of authenticity which can capture some paradigmatic cases and intuitions. To my knowledge, no analysis of MMTs in terms of a dual-basis, process view of authenticity has been provided in the literature so far.^7^ Looking beyond what changes MMTs may bring about to the fact that they bring about change *by means of memory modification* can provide novel insights into potential threats and chances memory modification offers for authenticity that can complement the existing literature on authenticity and MMTs.

## What Could Memory Modification Look Like?

To assess the impact of MMTs on a process view of authenticity, the specifics of how MMTs may eventually work are crucial. MMTs could be used to modify different memory systems but since episodic memory is particularly relevant for identity and authenticity [33], I will focus on the modification of episodic memories. The necessary and sufficient conditions for a mental state to be identified as an episodic memory and how we can distinguish it from imagination or semantic knowledge is an ongoing debate in philosophy and science of memory [61, 62]. The argument of this paper does not hinge on the specifics of the definition or taxonomy of episodic memory. Whether a specific mental state is best considered as semantic knowledge, imagining, or episodic memory is not relevant for authenticity. What matters is what and how we remember, in the sense of the content and phenomenology of our memories. Therefore, I will use the term “episodic memory” in a broad, everyday sense as making past experiences available at the present.

In the following, I present three levels to characterize MMTs: 1) which dimension of a memory trace is modified: the factual content, the sensory-like experience, or the autonoetic consciousness, 2) which kind of experience is modified: trivial or formative memories, short or long timespans, the memory of using MMTs itself, memories of a victim or a perpetrator, or debilitating memories, and 3) what the process of memory modification looks like: the time of intervention, the time to take effect, whether it is a one-time or a repeated procedure, and the method (e.g., a drug or optogenetics). With these distinctions, I aim at identifying a large scope of possible characteristics of MMTs which are relevant regarding their influence on authenticity.

1) We can characterize MMTs regarding *the dimensions of memory* they modify. Firstly, MMTs could affect *the factual content* of memories. They could erase a certain episode, for example, if Lady Macbeth would delete all memories of how she tried to convince her husband to murder the king, or only parts of it, such as specific objects, persons, emotions, or general details (leading to a fuzzy memory). Furthermore, the factual content of a memory trace could be modified by introducing wrong elements. This kind of editing could affect what occurred in a situation as well as what the person thought or felt in that moment. MMTs could also be used to bring back true but forgotten content.

Secondly, *the sensory-like representation* of memories could be modified. Some of our memories conjure a representation that is similar to regular sensory experiences. I may remember having dinner with a friend last week and see myself sitting at the table, almost taste the food, and feel how happy I was to see her. Other memories only consist of semantic knowledge (it is a matter of debate if this should count as a memory [61]). I know *that* I went camping when I was seven but I cannot visualize it anymore. Through MMTs, this sensory-like representation accompanying some memories could be reduced, erased, intensified, or retrieved.^8^ To use Erler’s example [1], after using a sensory-like representation dampening MMT, Liz still remembers that she used to be bullied at school and that it made her sad and angry, and she knows what occurred in specific episodes when she was bullied. But she no longer has a mental image of it or feels the sadness and anger she experienced.^9^ Besides affecting the sensory-like experience as a whole, MMTs might modify it only selectively, by erasing, lowering, increasing, or bringing back the detail and intensity of, for instance, only the emotional aspects without affecting visuals and other sensory representations.

Third, MMTs could erase the *autonoetic consciousness* of a memory.^10^ Reliving an experience can be understood as having a sensory-like experience that is accompanied by autonoetic consciousness. Endel Tulving introduced the characterization of episodic memory via the autonoetic consciousness, a unique kind of subjective experience during retrieval [63–65]. Tulving argued that episodic memories are accompanied by a directly-given feeling of reacquaintance or reexperience, which he referred to as autonoetic consciousness. There is a familiarity to the scenes revisited in episodic memory as well as an awareness that this happened to oneself. Stanley Klein and colleagues presented the case of R.B. who exhibited an unusual memory disorder after an accident [61, 66]. His memories lacked autonoetic consciousness: “I can see the scene in my head…I’m studying with friends in the lounge in the residence hall. But it doesn’t feel like its mine…that I own it. It’s like imagining the experience, but it was described by someone else.” ([61], 18) Without the autonoetic consciousness, it does not *feel* as if the event occurred in the past to oneself. But it seems possible to retain the knowledge of the content of the event, including the knowledge that it occurred to oneself, as well as a sensory-like representation. R.B. knows that the episode in the residence hall is one of his memories (if we still want to call it a memory) in the sense that he knows it occurred in his past. It just no longer feels like a memory. MMTs could be used to cause the same kind of detachment from one’s memories. By referring to the autonoetic consciousness I do not intend to adopt Tulving’s typology or definition of memory. The sensory-like experience and the autonoetic consciousness (or feeling of familiarity) are commonly accepted phenomenological qualities of memories [62, 67, 68]. Even if only some episodic memories are accompanied by autonoetic consciousness or a sensory-like experience, these phenomenological qualities of memories could be targeted by MMTs.

Before turning to the next level of characterization, I want to address the notion of memory dampening. Memory dampening is usually described as “lowering the emotional impact of memories” or “easing the ‘sting’ of bad memories” [7]. Memory research suggests that it is possible to manipulate the emotional content of memories independently of their factual content [10]. Memory dampening is a much-debated issue in neuroethics because some drugs, in particular Propranolol, showed some success in reducing the vividness of traumatic memories [11–13]. Propranolol has, for instance, been successful in lowering the startle response of previously conditioned fear memories while leaving the memory of the connection between the stimulus and the shock intact [9]. However, it remains unclear how individuals do (in cases where Propranolol showed success) or would (regarding other, future applications and technologies) experience emotionally dampened memories, particularly in case MMTs are applied to rich, autobiographic memories. There are various options of how one could potentially lower the emotional impact of memories. MMTs could affect the factual knowledge of how the individual felt during a certain event. They could erase or lower the intensity of the sensory-like representation of the emotion or the mental image in general while retaining corresponding factual knowledge. Memory dampening could even occur by removing the autonoetic consciousness. All of those options could potentially lower the emotional impact of memories. As I will argue, which one is used matters for authenticity.

2) Memory modification can further be used to change different *kinds of experiences*. Of course, MMTs may be used to modify all sorts of experiences. The different experiences I distinguish in the following are not meant as an exhaustive list, but they are particularly important in the context of ethical concerns about memory modifications. Hence, this category is more heterogeneous than the previous and next one. MMTs may affect formative (or self-defining) memories, which play a central role in a person’s self-narrative and which shape the trajectory of their life and their self-image, or trivial memories, such as what you had for breakfast. It seems rather unlikely that memory modification will be applied to trivial memories but what is trivial for one person may be important for another so there may be external reasons for modifying trivial memories. We may modify short timespans of maybe only a few minutes or hours or long timespans of weeks, months, or even years. Another kind of memory potentially affected by MMT would be the memory of performing a memory modification itself. One could forget having used an MMT at all. The memories of a situation of victimization or of a situation in which the individual was a perpetrator are further kinds of memories that may be modified. This is particularly important because a promising future application of MMTs is in the treatment of PTSD, which is often caused by a situation with a victim-perpetrator dynamic. Lastly, MMTs will likely be applied to memories that are debilitating, such as memories causing PTSD.

3) Finally, I want to shed some light on possible ways of what *the process of memory modification* may look like. Research in memory science suggests that there may be different possible times of intervention. MMTs could be applied *before* the situation that is being modified, to stop the memory trace from turning into a solidified memory in the first place. First responders could, for example, use MMT before entering a potentially traumatizing situation. They could be applied *shortly after* the experience or *after a long time*, even weeks, months, or years later, by affecting memory reconsolidation. The latter case is particularly relevant for individuals who want to use memory modification after having developed PTSD. Memory modification could furthermore occur gradually or instantaneous; it could require a one-time intervention or a repeated procedure (e.g., daily medication); and it could be realized in the form of a drug, optogenetics, a talk therapy, electrical stimulation, psychosurgery, or other, as of yet unknown, methods.

There may be other relevant characteristics of memory modification but this overview should give an impression of some of the most important options of how memory modification could work. Before moving to the impact of MMTs on authenticity, I want to draw a brief comparison to regular forgetting. How exceptional MMTs will be, depends on the specifics of memory modification. However, many of the potential outcomes of memory modification could also occur through normal forgetting. Regular cases of forgetting occur in numerous disparate ways. Hence, it is unlikely that MMTs will substantially differ from all manners in which we normally forget. Nonetheless, we can identify a distinguishing feature of MMTs: we can *directly and intentionally control* our forgetting. Normally, we just happen to forget things, without us actively trying to do so. It is also possible to support regular forgetting to some degree. For example, after a break-up, a person may remove objects that can trigger memories, or he may try to distract himself every time an unwanted memory pops up. Eventually, this could contribute to a state in which he would no longer remember even in the presence of a trigger since repeated remembering strengthens memories [69]. But those are only indirect measures to make it easier for him to forget. A reliable and successful method for memory modification would provide us with direct and intentional control over our forgetting. The memories can be targeted directly and not only their triggers or our reaction to them. The so-far paradoxical and futile direct attempt to forget, such as when Kant wrote himself the note: “The name Lampe must be entirely forgotten.” [70], would be possible with memory modification.

## Authenticity: a Narrative Account

To analyze MMTs based on a dual-basis process view of authenticity, I suggest a narrative account of authenticity which can accommodate both criteria. Contra Andrea Lavazza [4, 71], my account should show that authenticity does not presuppose a notion of “rigid identity” but can be defined on the basis of a dynamic, narrative concept of the self. The guiding idea behind narrativism is that we experience our lives as an internalized, evolving story [34, 35, 72]. This self-narrative organizes and shapes our experiences by connecting them to our past and future. Through it, we can make sense of our actions and ourselves. The self-narrative is formed in interaction with others and in reaction to their narratives about ourselves as well as shared cultural narratives [33]. The narrative self is a diachronic, ordered perspective we take towards ourselves and our life which allows us to engage in person-specific practices if certain conditions are met [35]. In philosophy, narrativism is rooted in the personal identity debate [34, 35, 73–76]. Thus, it is not surprising that in the neuroethical debate, narrativism is typically discussed in the context of personal identity and not authenticity (or at least not in a sense which draws a clear distinction between identity and authenticity) [36, 77–83].

To assess the influence of memory modification on authenticity, I rely on a narrative account of authenticity that I have introduced elsewhere [56]. In the following, I briefly summarize the account, but the details should become clearer in the discussion on memory modification. To be authentic, a person needs to have and act in accordance with a self-narrative that is *sustainable*, meaning it accurately and coherently represents one’s lived experiences, and that depicts a *well-defined* person. A sustainable self-narrative is not in tension with one’s lived experience. Therefore, it does not require too much mental work to uphold, such as implausible rationalizing or twisting, ignoring, and suppressing of experiences. Relevant facts about one’s subjective experience (e.g., emotions, thoughts, or intentions) and relevant objective facts (e.g., body features, nationality, or facts about actions and life events) are adequately, coherently, and intelligibly represented in the self-narrative. A person who considers herself helpful but always refuses to help others when the opportunity arises would have to ignore or rationalize the fact that she repeatedly acted unhelpful. To uphold a self-narrative representing her as helpful, she has to try to fit the narrative to a reality that does not support it, which eventually renders it unsustainable. This lack of self-transparency and truthfulness with respect to her lived experiences would make her less authentic. A sustainable self-narrative acknowledges who you were in the past as well as the boundaries of who you can be in the present.

The sustainability condition combines elements of the reality and the articulation constraint introduced by Marya Schechtman in her narrative account of personhood [35]. The constraints are similar because personhood and authenticity are closely interconnected but the motivation behind introducing the sustainability constraint for authenticity and the reality and articulation constraints for personhood is different. Schechtman argues that the reality and articulation constraints are necessary because personhood is tied to practices involving other persons with whom we have to (more or less) agree on a shared reality and whom we occasionally have to explain ourselves to [35]. In contrast, the motivation behind introducing the sustainability constraint is that authenticity requires self-knowledge and self-discovery. The ideal of authenticity emphasizes the acknowledgment of and truthfulness to who you are on a subjective and objective level. Thus, while the building blocks are similar, they are used for different ends – in this case, for an original account of narrative authenticity. Personhood requires these building blocks to ensure a certain degree of transparency and truthfulness on an interpersonal level whereas the ideal of authenticity requires truthfulness and transparency on a self-relational level, as suggested by both essentialist and existentialist accounts of authenticity.

The second condition is that an authentic person is well-defined. The question of who you are should have a reasonably clear answer and not multiple disparate and equally right ones. If a self-narrative is overly vague and relies too heavily on potentiality and externalization, the self is not sufficiently well-defined to be authentic. If your life just happens to you and you do not shape it through decisive actions or if you define yourself via things that could have been or might be your identity is elusive. It is not clear what kind of person you are. It remains a matter of speculation how you would have acted or will act, given the chance. Self-directed actions can confirm and define self-narratives. An example of a lack of self-definition would be a person who centers his identity around the idea that he really is an exceptional author, but unlucky circumstances have prevented him from writing a masterpiece. As long as he does not act on his alleged literary talent, it remains unclear whether he really is the person he takes himself to be. Not every case of self-definition through potentiality and externalization is problematic but if they are central elements of the self-narrative that remain unactualized, the person fails to sufficiently define himself and create a fact of the matter about who he is.

One may worry that the sustainability condition for authenticity poses unrealistic demands since extensive false memory research shows that we are often mistaken about our own past [84]. Moreover, our efforts to keep our self-narratives coherent probably contribute to distortions of our memory [85, 86]. We can make up all kinds of stories to try to make sense of our lives. Extreme examples are cases of people suffering from Korsakoff Syndrome who have extensive amnesia and who are caught in a constant effort to make up stories explaining their current situation [87]. The sustainability condition is meant to set boundaries to ensure the standards of authenticity for self-knowledge and self-discovery are met but also leave some leeway since normal levels of forgetting and misremembering should not prevent a person from being authentic. The self-narrative should be accurate to a degree that ensures that it is not implausible or unintelligible because we ignore or suppress important information about ourselves. If inconsistencies build up and tensions with other sources of information about oneself (e.g., documents, photographs, accounts from other people, or bodily indicators) accumulate the self-narrative is no longer helpful for navigating life and thereby no longer sustainable. A highly inaccurate self-narrative hinders us, for example, in carrying out plans, interacting with others, or predicting the future. Minor mistakes in remembering the past which do not carry much explanatory weight in one’s life story are not problematic for sustainability.

Narrative authenticity is a dual-basis view because the construction of a self-narrative combines elements of self-discovery and self-creation. On the one hand, we have to know or discover various subjective and objective facts about ourselves which feature in the self-narrative.^11^ To have a self-constituting and authentic self-narrative, one has to acknowledge the facticity of the human being that takes this narrative perspective. This includes knowing or discovering one’s more stable characteristics, bodily features, or facts about life events. The self-narrative constitutes the self, but it cannot do so independently of human facticity. On the other hand, understanding one’s trajectory through life in the form of a self-narrative involves creative elements. Based on the same brute life facts we can construct different stories about a person. Different moments can be seen as central, different actions can be embraced or rejected, and different overarching goals can be identified. Moreover, according to the self-definition criterion, we should actively shape ourselves and our future which can be understood as an act of self-creation.

Furthermore, the narrative authenticity account is a process view because changes in one’s personality, character traits, or one’s likes and dislikes are not understood as detrimental to authenticity in and of themselves. Narrative identity is inherently dynamic. Self-narratives change by adding new elements, such as new events, beliefs, or traits, as well as by reinterpreting the past. In addition, for a self-narrative to be coherent, personal change is sometimes required. We can imagine a person undergoing an experience after which the self-narrative would become incoherent if the person did not change aspects of her identity. The kind of person she is according to her self-narrative would change after going through such an experience. Changing one’s narrative identity, even if it occurs quickly and pertains to central aspects of one’s self-narrative, can be a natural consequence of maturing and making new experiences. Aging usually leads to changes in personality or transformative experiences. Narrative authenticity acknowledges the dynamic nature of our identity. What matters for authenticity is whether the change can be intelligibly and coherently integrated into the self-narrative as well as whether it contributes to self-definition.

Catriona Mackenzie and Mary Walker [80] and Françoise Baylis [88] have argued that “First, the authenticity framework is conceptually misleading insofar as it fails to carefully demarcate authenticity as self-discovery from authenticity as self-creation. Second, each of these conceptions of authenticity is flawed insofar as authenticity as self-discovery wrongly presumes a static inner life and authenticity as self-creation blurs an important distinction between identity and autonomy. Third, both of these conceptions of authenticity fail to properly account for the dynamic, relational nature of identity.” ([88], 368). The narrative account of authenticity stands as a counterexample to this critique. I have argued that it is possible to construe an account of authenticity which combines elements of self-discovery and self-creation. Authenticity can be understood as an ideal that provides guidance between these two kinds of self-relation. My account does not presume a static inner life but is based on a narrative self view which acknowledges that identity is dynamic and relational. Moreover, it upholds the distinction between identity and autonomy. According to the narrative authenticity view, following autonomously chosen principles can be inauthentic. A person who suppresses other important aspects of the self in favor of her autonomous choice would contradict the sustainability condition and even autonomous choices may not be self-defining if they are overly reliant on externalization (for more on the distinction between autonomy and authenticity see [56]).

## Memory Modification and Authenticity

In the following, I analyze the different possible characteristics of MMTs identified in the third part in light of narrative authenticity. While I do hope to capture common intuitions about authenticity with my account and to ground them in a theoretically and empirically well-founded theory of the self, not everyone will agree that this is how we should understand authenticity, a concept notoriously difficult to define. The following proposal of how memory modification influences authenticity should however also be informative for understanding MMTs in terms of other accounts of authenticity, particularly if they also include norms of self-knowledge and self-definition.

### Dimensions of Memories

The possibly most far-reaching form of memory modification is the deletion or editing of the factual content of a memory. Memory deletion (of timespans or components of memories such as emotions or objects) can lead to a less accurate and coherent and thereby to a less sustainable self-narrative. The deletion of a memory trace can create a gap in the self-narrative, if important, self-defining memories are affected. It can be difficult to adapt to this distortion if the deleted memory carried some explanatory weight about who you are and what occurred in your life [8]. If you no longer know where you were or what you did in a certain period of your life you may no longer understand how you ended up where you are, why you acted a certain way, or why you care about something. Your life story makes less sense and you may become less intelligible to yourself.

Memory research suggests that characteristics can be preserved independently of the memory that contributed to their formation [89]. Episodic remembering of how we acquired traits or how we acted on them is not necessary to keep having or knowing about them. But our understanding of our traits or values and thereby our understanding of ourselves necessary for authenticity is not completely independent of our memories [90]. Liz may still care for the anti-bullying campaign she established, even after erasing the memories of how she herself was bullied, but she lost her knowledge of one of the reasons for why she cares.^12^ Thereby, her self-narrative loses coherence and by that sustainability and authenticity.

I do not want to imply that the self-narrative would necessarily fall apart and lose all its meaning and coherence by deleting or blurring some memories or mental images (compare [7]). It would become less coherent and gradually reduce authenticity but to become a substantial threat to authenticity, the deleted memories would have to carry explanatory weight in relation to one’s identity. Furthermore, by erasing memories, I may create a discrepancy between what others know about me and what I know. Narrative self-constitution is not done in isolation but in dialogue and negotiation with others. We have to deal with the stories others tell about ourselves. By deleting the own perspective, we can no longer contradict the views of others or contribute to a fuller picture [91]. Thereby, the self-narrative becomes more prone to inaccuracy and manipulation.

Extensive memory deletion can also leave the self less well-defined, especially if it pertains to formative memories or long periods. Throughout our lifetimes we generate facts about who we are. By deciding on a career, how to interact with friends, family, and strangers, or by choosing whether to walk or take the bus, we create facts about ourselves. All those actions and choices as well as the things we experience without our direct influence (e.g., being in a car accident) are sources of self-defining facts. With time, our self-narratives grow richer, more detailed, nuanced, and substantial and the self becomes more well-defined. By erasing memories, the self-narrative is being diminished. In extreme cases of memory deletion, a person could become insecure about who he or she really is. Of course, erasing memories of events does not erase the occurrence of the events. However, according to the narrative self view, the self is constituted through an active, dynamic, and evolving interpretation of one’s life trajectory. Whether or not Liz remembers being bullied is decisive for whether it is part of her self-narrative. Her remembrance is critical to whether this experience will continue to shape the way she interprets, evaluates, and engages with the world. Moreover, it seems that to truly erase the memory of an experience, it would be necessary to additionally modify possible instances of remembrance, reflection on, and reliving of those moments and it may affect numerous other memories which are closely connected to the target memory. Thus, in some cases, even the deletion of one episode of one’s life may require substantial memory modification and lead to a relevant reduction of self-definition.

The introduction of false memories would reduce the accuracy and most likely also the coherence of the self-narrative. Interestingly, they could however also make the self-narrative more coherent, depending on how the introduced memories fit with the rest of the self-narrative. Nonetheless, the individual would be deluded regarding his past which could render the self-narrative less plausible in the light of possible contradicting evidence about the modified episode. Furthermore, the choice to delude oneself through memory modification by introducing false memories goes against the norm of authenticity to know oneself and to have an accurate self-narrative, even if no external evidence would challenge the modified self-narrative.

Enhancing memory or bringing back true memories could in some cases lead to a more sustainable and well-defined self-narrative. However, not every kind of memory enhancement is necessarily beneficial for authenticity. Just as memory deletion is most threatening to authenticity if memories are affected that carry some explanatory weight within the self-narrative, memory enhancement would be beneficial if this kind of valuable information would be brought back or enhanced. Remembering more irrelevant details would not be problematic for authenticity (unless it would in some sense crowd out a person’s memory) but it would not be beneficial either. Striving for authenticity does not mean one should seek the most detailed self-narrative by adding more and more information about the course of one’s life. In a self-narrative that is meaningful and self-defining, we identify some goals, values, beliefs, moments of our life, or other characteristics as more important than others, connect a series of moments to an overarching event or a life period, group similar events together to a kind of events experienced multiple times, and find patterns in our life-experiences and our behavior [92]. All of this involves abridgment, summarizing, and selection. This is necessary for the self-narrative to fulfill its functions of making ourselves intelligible and of giving meaning to our experiences [34, 72]. A person may remember every detail of every interaction with his father, but he may still have less self-knowledge than a person who forgot most of them but recognizes, understands, and remembers the patterns in their interactions, how he tends to react, and why. Processing experiences and transforming them into long-term memories can entail clearing them of unnecessary detail and connecting and enriching them with further semantic knowledge. Thus, the ideal of authenticity does not demand excessive knowledge of the details of one’s life but a profound understanding of relevant episodes and characteristics.

In cases where MMTs would reduce or remove the sensory-like representation of memories, authenticity is likely not affected. The factual content of our memories can uphold the coherence, accuracy, and self-definition of the self-narrative, even without a sensory-like representation. Even if Liz does not conjure up a mental image of the faces of her bullies, she still knows that she was bullied and she can still understand why she resented them afterward and why she founded an anti-bullying campaign. As long as she retains the relevant facts about what occurred, how she felt, and what she thought, the sensory-like representation may be modified without detrimental effects for authenticity. It is however likely that the memory will be less detailed in that case. When we conjure mental images of our memories, we can “search” them for factual content we have not yet been consciously aware of. I may play a scene of a day in the park in my head to find out if my friend was drinking a coffee while we walked or if dogs were running around. Removing or reducing the sensory-like experience would likely reduce our access to this kind of information. However, as argued above, especially the small details of our memories are often not necessary for sustainability and self-definition.

The manipulation of the autonoetic component of memories does not affect the coherence of the self-narrative either. As the example of R.B. shows, even if the sensory-like experience is not accompanied by a feeling of reacquaintance, the individual can still retain the factual knowledge that what he remembers occurred to himself. R.B. knows that it was him studying with his friends in the lounge, even if while remembering, it does not feel that way to him. However, it seems that memories that lack an autonoetic component are in some sense less integrated into the self-narrative. A memory that is not experienced as mine does not shape and organize my experiences in the same way as one that has this phenomenology of “mineness”. R.B. reported that when he had to relearn how to walk after his accident, he did not feel down or experience a sense of loss: “Because it was as if I was learning to walk for the first time. […] I knew that I once could walk, but it wasn’t ‘me’ who once could walk” ([66], 688). For the self-narrative to provide the first-person perspective through which we evaluate, interpret, and engage with the world, as understood in the narrative self theory, the autonoetic component of memories is required. The memories of the time before R.B.’s accident which lack the autonoetic component are still a part of his self-narrative, insofar as they can help him make sense of how he ended up in his present situation, such as why he has certain skills or a degree from MIT, but they do not shape his experience in the same sense as other memories. The lack of ownership or “mineness” does not seem to pose a direct problem for either sustainability or self-definition and is, therefore, no threat to authenticity. R.B.’s self-narrative is still accurate and coherent and there is no confusion or ambivalence as to who he is. Nonetheless, having memories that are not felt as one’s own can be a disconcerting experience, as in the case of R.B., and may cause other issues for identity and narrative self-constitution. They are, however, beyond the scope of this paper.

### Kinds of Experiences

After discussing the possible dimensions of memories that could be affected by MMTs, we may now look at the kinds of experiences that could be modified. It should be fairly intuitive that the impact on narrative authenticity would be less pronounced in case trivial memories or short time-spans are affected compared to formative memories or long periods of one’s life. Erasing what I had for breakfast will have less of an impact on authenticity than erasing the past 10 years of my life.

Retaining the knowledge of using MMT can mitigate negative impacts on the coherence of the self-narrative. If individuals remember that they used an MMT, they can account for the explanation gap created by memory modification. They know why some of their actions or views are not fully intelligible to themselves. Liz may understand that she founded an anti-bullying campaign because of an experience she deleted. The knowledge of using MMTs can at least make sense of the distortion of the self-narrative even if it does not dissolve it.

Furthermore, there seems to be a relevant difference between modifying the memories of a victim and a perpetrator. There is a qualitative difference in how *what we do* defines us compared to *what happens to us*. The latter can play a central role in the self-narrative and drastically shape the trajectory of one’s life. Moreover, acknowledging, understanding, and finding meaning in sufferings can be crucial to cope with them and to develop a self-narrative that helps to navigate the world [93, 94]. However, actions can be self-defining on a further level compared to passive experiences. Both feature in the self-narrative as occurrences in one’s life but on top of that, actions can express and define views, beliefs, values, and other characteristics of a person. If Lady Macbeth erases the memory of how she helped to murder the king, she deletes a self-authored action that is defining her as a person. This action is telling us and herself not just something about what happened in her life but about what she is capable of as well as her values, goals, and overall character. It is fundamentally defining who she is. A victim of bullying who erases the memories of those episodes is first and foremost erasing something he or she suffered. Of course, things are not so simple, since how one reacts to an experience can also be self-defining and active. Nonetheless, for victims, it seems to be possible to integrate the experience into the self-narrative as something that occurred to them in which they did not take an active, self-directed part, whereas this is not an option for a perpetrator. Therefore, modifying the memories of a victim can, in some cases, be less threatening to self-definition than modifying a perpetrator’s memories.

In the case of a modification of debilitating memories, MMTs can help individuals to more actively lead their lives and to actualize potential characteristics. Thereby, they can contribute to a more well-defined self-narrative. For example, a person could think of herself as being helpful but PTSD leaves her so drained of energy and focus that she cannot actually help anyone. To be a well-defined person she should eventually actualize this alleged helpfulness [56]. MMTs could help her to achieve this. A further possible positive effect of MMTs on authenticity is that they could help some people to integrate traumatic experiences into their self-narrative. Reducing the emotional weight of memories might, in some cases, facilitate the integration of traumatic experiences into the self-narrative. Truths that might have been hard to face can become more manageable [3]. Thereby, the self-narrative could become more accurate, coherent, and sustainable than before the treatment.

### The Process of Memory Modification

Lastly, I want to discuss the possible effect of MMTs on narrative authenticity regarding the process of how memory modification could be actualized. The more time passes between the experience that is being modified and the modification itself, the higher the disruptive potential of MMTs. Experiences and the accompanying memories can influence the course of a person’s life drastically. After a long period, the transformations caused by this experience accumulate. Years after having been bullied, Liz may, for example, found an anti-bullying campaign. In such a case, a late memory modification can disrupt the self-narrative and make present goals, views, values, and plans less intelligible. At an earlier period (for example, just after Liz left school and had not yet founded the anti-bullying campaign), fewer decisions had been influenced by the experience. Hence, the memory modification would be less disruptive. In the case of a preventive memory modification, the memory modification would only disrupt the coherence of the modified experience itself. The person would not know what they did or how they felt at that time, but no long-term decisions would be affected.

The focus on the coherence and intelligibility of the self-narrative can provide an answer to the puzzle raised by Jesse Gray [27] on the temporal component of memory modification. Gray raised the question why it seems more disruptive to authenticity if Liz’s memory is modified years after the bullying incident when she had already founded the anti-bullying campaign, compared to an immediate modification just after the event since in both cases it would concern self-defining memories. If authenticity does not hinge on whether the self-narrative changed by altering self-defining memories (as argued by Zawadzki and Adamczyk [8]) but on whether it remains intelligible, accurate, and self-defining, we can understand why the time of intervention matters for authenticity without having to claim that Liz’ memory of bullying was not self-defining in case she modified the memory immediately after the experience.

A gradual memory modification would be less disruptive compared to MMTs that take effect instantaneously. Gradual changes in memories allow for an ongoing adjustment of the self-narrative. The individual is not suddenly left with possibly unintelligible and free-floating values, beliefs, or goals but has time to adjust by grounding the values, beliefs, goals, etc. in other ways or by shifting the focus of what is central in her life to something else. Through a gradual change, the self-narrative slowly accommodates the modified memory which can mitigate the loss of coherence.

In case of a repeated procedure for memory modification, such as a daily drug intake, the person would most likely retain the knowledge of using MMTs. As discussed above, this could lessen the impact on coherence. Whether the technique for memory modification is, for example, a drug, a talk therapy, or genetic engineering only matters for authenticity insofar as it may determine other characteristics of MMTs discussed above, such as whether it takes effect gradually, whether it is a repeated procedure, or which kinds and dimensions of our memories it can modify.

## Shifting the Balance

Before discussing the value of authenticity and concluding, I want to address a fundamentally different kind of impact memory modification can have on authenticity. MMTs could shift the balance between self-discovery and self-creation within authenticity. The concept of authenticity combines assumptions about the nature of the self and norms regarding the self. Most current accounts of authenticity, notably those which understand authenticity as combining self-discovery and self-creation (including the narrative account used in this paper), assume that there are things about us we can hardly change and others that are up to us to define. The ideal of authenticity introduces norms for both: we have to discover and acknowledge some aspects of our identity and we have to actively define ourselves. On the one hand, a person cannot just decide, for instance, not to be shy. To be authentic, this person would have to acknowledge and accept his shyness, even if he rejects and tries to change it. On the other hand, you should actively take your life in your hands to shape and define yourself. Taken together, to be authentic you should actively choose a life path within the boundaries of who you can be.

Memories of the past are constitutive of the boundaries we find ourselves in today. The shy person, for example, might be shy due to a lack of confidence because he did not feel supported in his childhood. With MMTs, the self becomes more malleable since we can manipulate those boundaries and open up new ways of who we can be. Because of this potential for self-change through MMTs, memory modification has been discussed as a means for moral enhancement [71]. Lavazza argues that a person who behaves anti-social or egoistic because she lost trust in other people after a traumatic event could be morally enhanced by modifying the memory of the trauma. So far, our memories have not belonged to the category of things we can control about ourselves. MMTs could change that. The boundaries of who we can be, which are usually almost impossible to change (or at least only through hard work, extreme circumstances, or after a long time), would become more malleable. Through MMTs, more of our characteristics can be accessed and shaped in an act of self-creation. But not only MMTs can contribute to this shift. Other neurointerventions, such as psychopharmaceuticals, neuroimplants, or psychosurgery, can also give us the ability to change ourselves in unprecedented ways. Future scientific and medical advancements will continue to push the possibilities for self-creation. However, even if you can freely choose every aspect of who you are, the ideal of self-discovery does not lose its relevance.^13^ You could still discover and acknowledge who you are and what you want and there might nonetheless be an appeal to reacting with gratitude and acceptance instead of creative self-change. But self-discovery and acceptance would no longer be the only possible reactions to previously unchangeable characteristics.

For some people, the only authentic paths through life which are available are very difficult. Their characteristics may not be rewarded in the private or professional life of their society. By changing the constraints of who you can be, neural interventions open up the possibility for life paths that are easier and maybe happier while remaining genuine. The shy person would not have to force himself to appear outgoing when applying for a job favoring an outgoing attitude. He would just genuinely feel like being outgoing. However, this raises serious issues of conformity, which would contradict a further pillar of authenticity: that we should not bend to the will of other people to fit in but be ourselves. This would be unsustainable in case you would not acknowledge that you are bending to peer pressure, and it would lead to a lack of self-definition because it would be unclear how you would have acted if you were not pressured by others. According to existentialist authenticity, we should not get lost in the other but choose ourselves freely. Social structures and demands can create a conflict between a characteristic of a person and a goal she wants to reach, such as a conflict between shyness and finding a job. As long as it is very hard to change one’s characteristics, one may try to set different goals or to change social norms. With the aid of neurointerventions, it becomes possible to change the characteristics of a person more easily. Thereby, the burden of change may be shifted to the individual, even if we might do better to change society to allow for a happy and flourishing life for people with any kind of characteristics.

## The Value of Narrative Authenticity

It has been suggested that authenticity is of both intrinsic and instrumental value. Charles Taylor famously argued that authenticity is an ethical ideal that can guide us to lead a fulfilled, meaningful life [42] and various psychological work shows that authenticity is beneficial for wellbeing (for an overview see [95]). I agree with the argument of Jan Christoph Bublitz and Dimitris Repantis [24] that the conclusions reached on the value of authenticity only apply to the definition of authenticity the respective work is grounded on. Especially the psychological literature uses rather fine-grained definitions of authenticity. We should be cautious when applying their results to other conceptions of authenticity.

When we look at the notion of authenticity Taylor defends as an ethical ideal, we can however see that the narrative account would fit his definition. Taylor stresses that a valuable ideal of authenticity is both an individualistic enterprise of original self-discovery and self-creation (which may include opposition to social rules and values) as well as embedded in and dependent on the social context because it is bound to shared horizons of significance and because self-definition happens in dialogue with others ([42], 66). Narrative authenticity combines self-discovery and self-creation on the basis of narrative self-constitution which is deeply embedded in a social and cultural context. The sustainability condition requires on the one hand individualistic self-discovery and on the other hand understanding, reacting to, and integrating of the narratives others make about ourselves. Furthermore, in constructing a meaningful, well-defined self-narrative we make use of culturally available canonical biographical forms, life scripts, and masternarratives [33]. The ideal of authenticity Taylor defends is broad enough to cover different accounts of authenticity, including the narrative view offered here.

Moreover, many people care about authenticity (in a broad sense) and aspire to be authentic: it is a common topic in popular culture [96, 97], countless self-help books on helping to achieve authenticity are being written and sold ([43], 22–25), and for many patients, feeling authentic is an important criterion for a successful treatment [53, 98, 99]. With the narrative account, I hope to have captured at least partially what people who care for authenticity strive for. Most approaches to ground neuroethics in a normative theory agree that patient’s interests should be protected in one sense or another (for example, in Kantian ethics, utilitarianism, principlism, or care ethics). Since being authentic is in the interest of many patients, understanding how authenticity can be preserved and identifying potential threats is ethically relevant. Patients should be informed about the possibility of becoming less authentic through MMTs and they may require support to avoid or cope with a loss of authenticity. Furthermore, it implies that we should continue to investigate the impact of MMTs on authenticity both on a conceptual and empirical level.

## Conclusion

By employing a dual-basis, process view of authenticity, we can see that whether, how, and to what degree MMTs may be threatening or beneficial to authenticity depends on the details of how memory modification would work. Specifically, it depends on the dimensions of memories and kinds of experiences that are modified as well as the properties of the process of memory modification. By focusing on the distinction between memory dampening and memory deletion, important differences within possible forms of memory dampening have been neglected as well as other relevant characteristics of memory modification (for example, whether the memory of a victim or a perpetrator is modified or whether it is a gradual or instantaneous procedure). Because the impact of memory modification on authenticity depends on which kinds of experiences are modified (see also [100]), it is case-dependent and cannot be generalized. Nonetheless, we can conclude that some kinds of MMTs are much less threatening to authenticity than others. Particularly concerning are the erasing or editing of the factual content of memories, notably if they carry explanatory weight in relation to narrative identity. But the rapid advancements in science and technology are not only changing ourselves – they also influence our concepts and ideals. Making MMTs easily and widely available would increase our possibilities for self-creation which may change the ideal of authenticity itself.

## Data Availability

Not applicable.

## Footnotes

1 I am concerned with personal identity in terms of the characterization question, i.e., which characteristics can we rightfully attribute to a person. In contrast, the reidentification question is concerned with numerical identity, i.e., is a person at t_2_ the same as a person at t_1_ [35].

2 Others have included speculations on possible properties of future MMTs based on currently existing applied and experimental methods in their ethical analysis of MMTs [5, 8]. However, their work, while being very insightful, is strongly tied to what we currently know about MMTs and does not explore possible properties of MMTs which cannot be derived from empirical work so far, notably how patients would experience their modified memories.

3 Authenticity is sometimes used in the sense of truthfulness, as honestly expressing what one thinks or feels, or as akin to autonomy, that to live authentically means living in accordance with one’s “true” goals and values [101]. I focus on the dimension of authenticity which is unique to this concept – the interplay of self-creation and self-discovery – and which cannot be subsumed under autonomy or truthfulness.

4 The degree to which MMTs may change one’s personality is important to assess the severity of the impact on authenticity, but it is not decisive for whether the memory modification was authentic in the first place. A person who is manipulated to give up his most central life plans and values is less authentic than if he is manipulated to dislike apples, but his authenticity is diminished in both cases due to the process of change.

5 It could be possible to construe an amended version of a self-discovery account in terms of a process view or of a self-creation-based conservation view. However, some of the most distinctive features of authenticity as self-discovery (or self-creation) would have to be abandoned so it would no longer be a typical self-discovery (or self-creation) account.

6 I would like to thank an anonymous reviewer for pressing me on this point.

7 Erler’s true self account [1], for example, defines authenticity via a self-discovery based conservation view. Pugh et al.’s coherence account [37] used by Przemyslaw Zawadzki and Agniezka Adamczyk [8], is a dual basis view that combines a conservation and process view, and Matthew Liao and Anders Sandberg [5] use a wholeheartedness approach after Harry Frankfurt [47] which can be understood as a self-creation-based process view.

8 Inserting a wrong sensory-like representation seems impossible without also changing the factual content of the memory.

9 Without a sensory-like experience Liz could no longer re-experience the sadness or anger as she felt it in that past moment, but the memory could still have valence since semantic memory also evokes emotional responses [102].

10 Since we do not lose the autonoetic dimension of a memory except in very rare pathological cases I will not discuss the option of retrieving the autonoetic consciousness.

11 This does not necessarily require a high degree of self-reflection. This kind of self-knowledge may also result from a more implicit awareness.

12 At this point I refer to the modified version of Liz’s case as introduced by Zawadzki and Adamczyk [8] where she established and worked full-time in an anti-bullying organization because of her own experience of being bullied.

13 I would like to thank an anonymous reviewer for raising this point.
